# The role of platelets in the regulation of tumor growth and metastasis: the mechanisms and targeted therapy

**DOI:** 10.1002/mco2.350

**Published:** 2023-09-14

**Authors:** Kaili Liao, Xue Zhang, Jie Liu, Feifei Teng, Yingcheng He, Jinting Cheng, Qijun Yang, Wenyige Zhang, Yuxuan Xie, Daixin Guo, Gaoquan Cao, Yanmei Xu, Bo Huang, Xiaozhong Wang

**Affiliations:** ^1^ Jiangxi Province Key Laboratory of Laboratory Medicine Jiangxi Provincial Clinical Research Center for Laboratory Medicine Department of Clinical Laboratory The Second Affiliated Hospital of Nanchang University Nanchang China; ^2^ Queen Mary College of Nanchang University Nanchang China; ^3^ School of Public Health Nanchang University Nanchang China; ^4^ The Second Clinical Medical College Nanchang University Nanchang China; ^5^ The Fourth Clinical Medical College Nanchang University Nanchang China

**Keywords:** mechanism research, platelets, targeted therapies, tumor growth, tumor metastasis

## Abstract

Platelets are a class of pluripotent cells that, in addition to hemostasis and maintaining vascular endothelial integrity, are also involved in tumor growth and distant metastasis. The tumor microenvironment is a complex and comprehensive system composed of tumor cells and their surrounding immune and inflammatory cells, tumor‐related fibroblasts, nearby interstitial tissues, microvessels, and various cytokines and chemokines. As an important member of the tumor microenvironment, platelets can promote tumor invasion and metastasis through various mechanisms. Understanding the role of platelets in tumor metastasis is important for diagnosing the risk of metastasis and prolonging survival. In this study, we more fully elucidate the underlying mechanisms by which platelets promote tumor growth and metastasis by modulating processes, such as immune escape, angiogenesis, tumor cell homing, and tumor cell exudation, and further summarize the effects of platelet−tumor cell interactions in the tumor microenvironment and possible tumor treatment strategies based on platelet studies. Our summary will more comprehensively and clearly demonstrate the role of platelets in tumor metastasis, so as to help clinical judgment of the potential risk of metastasis in cancer patients, with a view to improving the prognosis of patients.

## Graphical abstract

The summary of interactions between tumor cells and platelets. ①Platelets can facilitate the tumor cells invasion. ②The interaction can cause inflammation, causing the decrease of NKG2D on the surface of NK cells. ③ Leukocytes help the EMT. ④In addition, tumor cells can cause platelet aggregation. ⑤These can help the tumor cells extravasation and angiogenesis. ⑥In the blood vessels, neutrophil extracellular traps (NETs) can stimulate the migration and invasion of cancer cells. Immune cells in the tumor microenvironment can release cytokines to promote the growth of cancer. The figure is made using Figdraw. The figure is Graphic Abstract image.

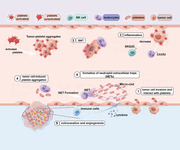

## 1 INTRODUCTION

Cancer cells’ genetic mutations and a large number of protein mutations allow them to proliferate uncontrollably. The ability of cancer cells to persist, proliferate, and even metastasize in the body is significantly related to their ability to evade recognition and attack by the body's immune system through a variety of mechanisms. Previous studies have demonstrated that the body's immune cells and fibroblasts can be used by cancer cells.^1^, ^2^, ^3^ A growing body of research suggested that platelets in the blood are also used by cancer cells to help them grow and become aggressive.

Cancer‐associated thrombosis is the second leading cause of death in patients with tumors.^4^ The incidence of venous thromboembolism has been reported to be 2%–20% and arterial thromboembolism within 1%–4.7% in patients with malignancy.^5^, ^6^, ^7^ Platelets, as important regulatory cells of thrombosis, play an important role in cancer‐associated thrombosis. Cancer cell‐induced platelet activation also facilitates the development and spread of tumors. Previous studies found that mean platelet volume and platelet distribution width are closely related to the prognosis of renal cell carcinoma, colorectal cancer (CRC), melanoma, and other tumors.^8^, ^9^, ^10^

**FIGURE 1:**
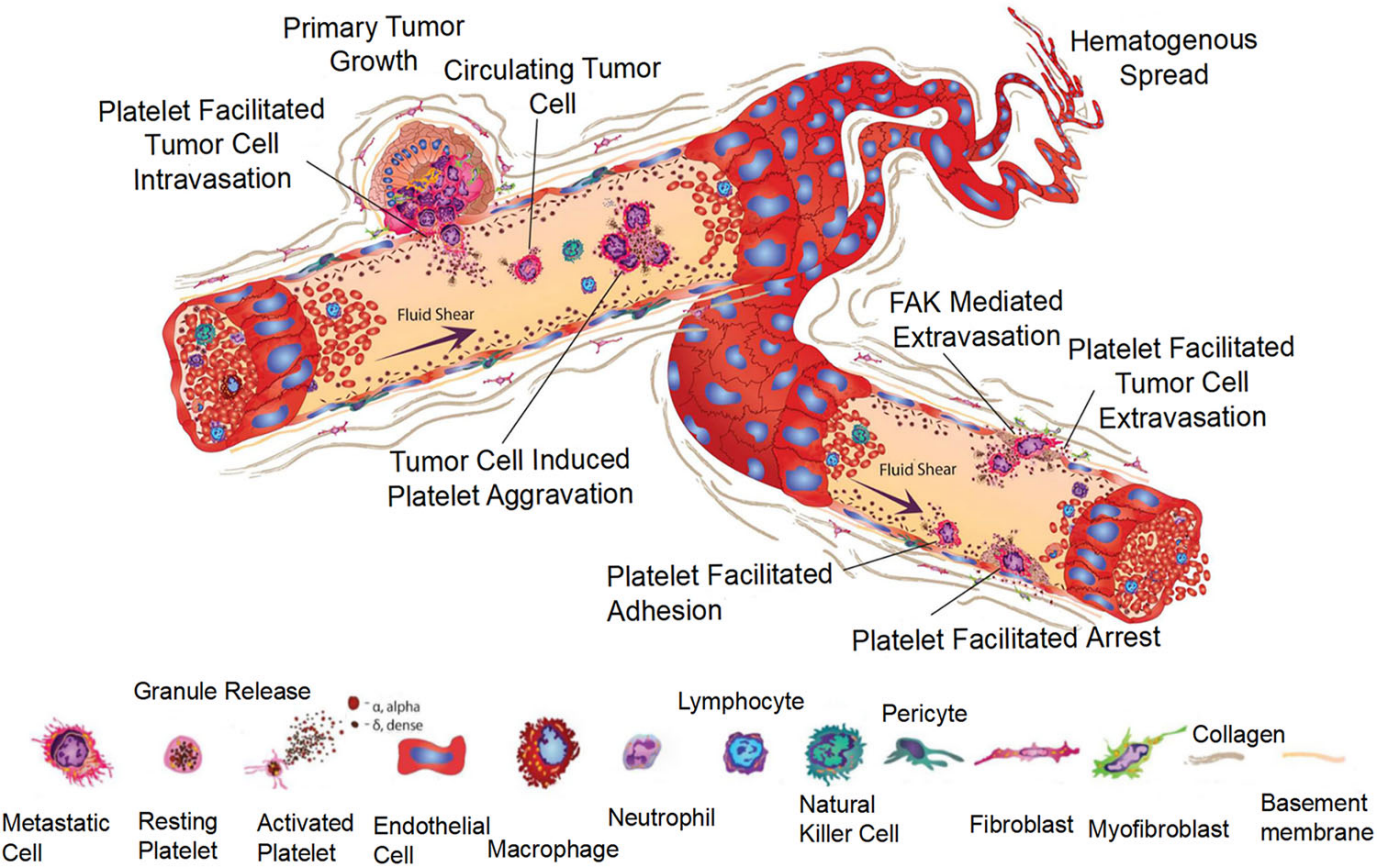
Presentation of the function of platelets in cancer growth and invasion. Platelets can release cytokines and other molecules to facilitate the growth of tumor cells. Reproduced with permission from reference 37.

Previous studies confirmed that tumor angiogenesis and tumor cell evasion of body immunity are closely related to the tumor microenvironment (TME).^11^, ^12^, ^13^ Numerous studies confirmed that platelets play an important role in tumor growth and metastasis,^14^ greatly influencing the behavior of cancer cells, while the physiology and phenotype of platelets are also affected by cancer cells. In the TME, platelets are activated and aggregated by tumor cells^15^, ^16^ and aid in the immune escape of tumor cells through paracrine secretion and direct contact, thereby increasing the survival of tumor cells during metastasis. Platelets interact with tumor cells through surface receptors and help tumor metastasis.^17^ The aggregation of platelets around tumor cells protects them from shear forces and natural killer (NK) cells in the blood, and provides a scaffold for tumor cells to adhere to the vessel wall. In addition, angiogenesis factors and growth factors secreted by activated platelets can promote tumor growth and angiogenesis,^18^ thus, establishing a good microenvironment for tumor metastasis. At the same time, tumor cells will further activate platelets and stimulate platelets to release active substances to optimize the growth environment of the tumor itself (Figure 1).^19^

Stromal cells and tumor‐infiltrating immune cells are the main cellular participants in TME. The tumor stroma is composed of different types of cells, including fibroblasts and endothelial cells.^20^ Remarkably, we can observe platelet infiltration into the tumor stroma,^21^ with increased blood platelet counts (PCs).^22^ Tumor‐infiltrating platelets can interact with other stromal players of TME. Platelets can release growth factors acting on neoplastic epithelial cells to trigger epithelial‐mesenchymal transition (EMT), and platelets interact with epithelial cells to promote angiogenesis. Activated platelets and their secreted products contribute to the formation of cancer‐associated fibroblasts (CAFs). These contribute to tumor promotion and progression.^23^ Platelets also adjust various angiogenesis regulators, which can turn on local angiogenesis in the TME.^24^ Moreover, intratumoral platelet accumulation is associated with tumor progression.^25^ In conclusion, platelets function as an important stromal component in the TME through interactions with other members.

Our article focuses on the function of platelets in the process of tumor metastasis, discusses the interaction between platelets and tumors, and more importantly, provides a reference for further research in this field by introducing the current platelet‐based tumor‐targeted therapies.

## 2 THE FORMATION AND CHARACTERISTICS OF PLATELETS

There are three main types of blood cells in human blood: red blood cells (the most numerous), platelets, and white blood cells (the least numerous). Platelets are generated in the bone marrow and circulate in the bloodstream. When bleeding or injury occurs somewhere in the body, they go to the wound and help stop the bleeding by gathering more platelets together with other clotting factors to form a clot.

Platelets are small, non‐nucleated cell fragments that originate from the largest megakaryocytes in the bone marrow.^26^ Megakaryocytes are the important regulatory cells in the hematopoietic stem cell microenvironment^27^ and are stimulated by thrombopoietin (TPO) to differentiate from hematopoietic stem cells in the bone marrow, which can migrate to the lungs to produce platelets.^28^ To avoid activation of platelets before they enter the circulation, megakaryocytes mature and undergo cytoplasmic remodeling to produce blunt projections, forming proplatelets. The proplatelet morphology elongates and thins over time, extending into the lumen of the blood vessel^29^ and remodeling into proplatelets in the blood under the shear stress of blood flow. Then, the proplatelets generate platelets by fission.^30^

Normal platelets are concave, oval, or disc‐shaped in circulation,^31^ and they are the smallest cells in the circulation. The number of platelets in the human body ranges from 150 × 10^9^ to 400 × 10^9^ per liter.^30^, ^32^, ^33^, ^34^ The outermost layer of platelets is coated with a variety of glycoproteins, which are either secreted by the internal granules of platelets or constitute the membrane components of platelets, and play a very important role in platelet adhesion and aggregation.^34^ The platelet membrane (PM) and the platelet contractile protein system are encapsulated underneath these glycoproteins, which help to maintain the platelet's disc‐like morphology. Apart from the membrane glycoproteins, there are many receptors on the surface of PMs. PM receptors that support hemostasis and thrombosis include the integrin gene family, the leucine‐rich glycoprotein gene family, the selectin gene family, and the immunoglobulin gene family.

## 3 INTERACTION BETWEEN PLATELETS AND TUMORS

In this section, first, we will discuss how platelets and tumors interact to promote tumor cell growth and immune system escape and later, we will focus on the mechanisms of their interaction in promoting the formation of new blood vessels and aiding tumor cells from the primary site through the epithelial stroma to the new site.

### 3.1 Tumor cell homing and tumor cell growth

As the neoplastic organism formed by abnormal proliferation of local tissues, tumor cells do not grow in isolation, but create a suitable “soil” for their growth, that is, TME, and promote their own development by interacting with a variety of cells in the body.^35^ Tumor cells and the microenvironment in which they reside are a functional whole. Tumor cells are regarded as seeds, while the microenvironment in which they reside is regarded as soil. Tumor cells and their microenvironment interact and coevolve to promote tumorigenesis.

Under normal physiological conditions, circulating platelets do not interact with inactivated endothelial cells. When cancer occurs, tumor cells release cytokines and growth factors that activate vascular endothelial cells, thus promoting the proliferation of the tumor cells. High expression of vascular hemophilia factor, P‐selectin, integrin protein, and tissue factor (TF) are activated by endothelial cells,^36^ which mediate platelet rolling along the endothelium by binding to adhesion receptors on the platelet surface. The interaction of platelets with activated endothelial cells induces complete activation of platelets and high expression of P‐selectin on their surfaces, allowing platelets to adhere firmly to the endothelium, thus facilitating local recruitment of platelets.^37^

**FIGURE 2:**
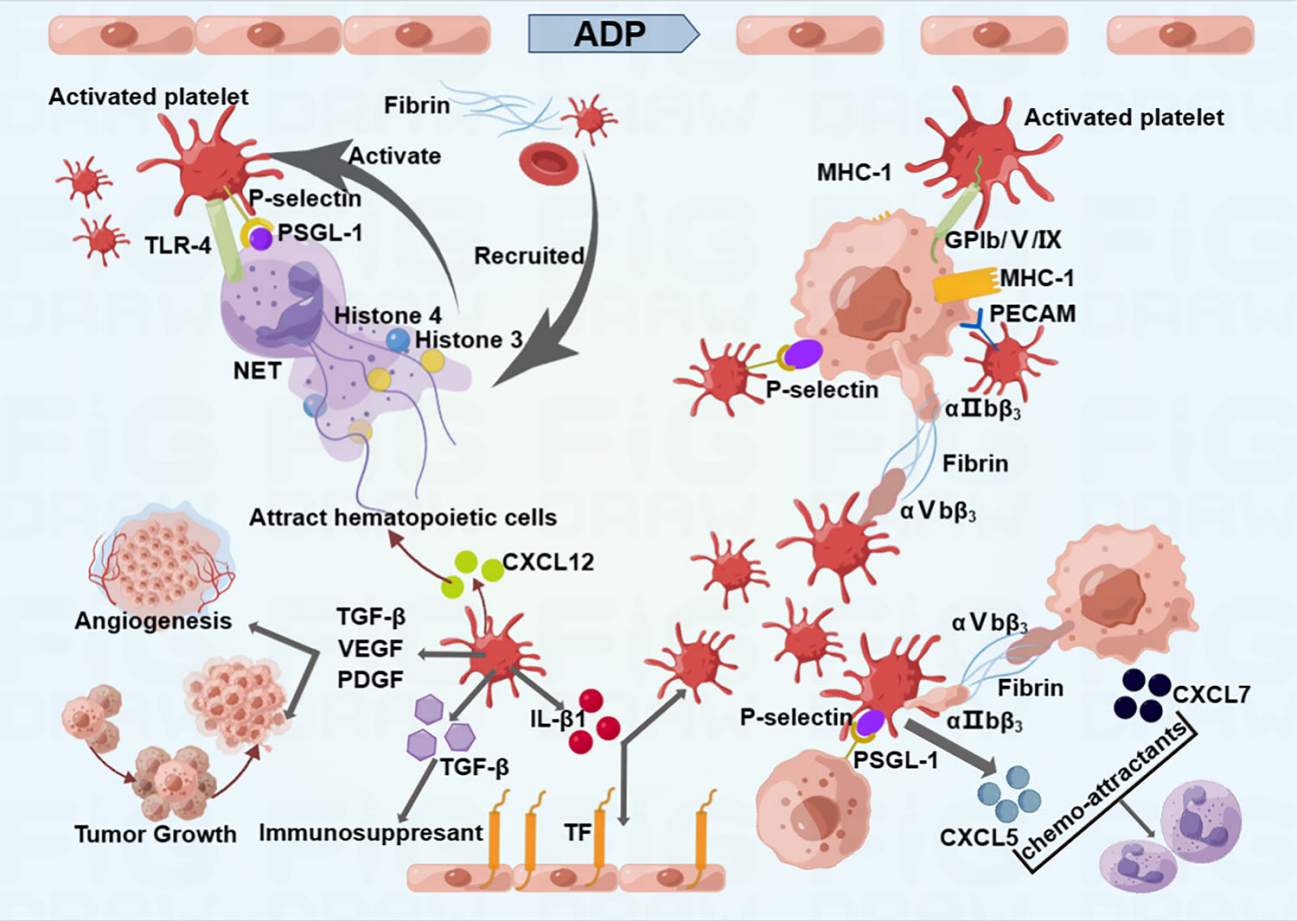
Various mechanisms of platelet activation. Platelets interact with tumor cells to produce inflammations in cancer. Reproduced with permission from reference 38.

Fully activated platelets release proangiogenic factors, which further activate endothelial cells, support angiogenesis,^38^ and regulate vascular permeability and vascular tone.^36^ At the same time, the proangiogenic factors, together with the activated factors stored inside platelets, are released into TME, further promoting revascularization and inflammation (Figure 2). For instance, lysophosphatidic acid (LPA), which is produced by activated platelets, can bind to LPA receptors (e.g., LAP1) on breast cancer cells and this interaction contributes to the proliferation and osteolytic bone metastasis of cancer cells.^39^ Apart from releasing contents, platelets also communicate with tumor cells via G protein‐coupled receptors on platelets’ surface and the loss of two critical G proteins (Gαi2 and Gα13) substantially reduced platelet extravasation and tumor growth.^40^ A large number of platelets are present in the TME,^41^ and these platelets help to build the TME by releasing platelet‐derived microparticles (PMPs), which support the overgrowth of tumors.^42^ While accumulating evidence indicates that PMPs are associated with poor prognosis in various cancers including those of lung and stomach,^43^, ^44^ PMPs also can induce apoptosis of solid tumor cells through transferring platelet‐derived RNA, primarily miR‐24.^45^ Mechanistically, platelet‐derived miR‐24 inhibited mitochondrial RNAs, resulting in growth impairment.^45^ Besides, interactions between platelets and tumor cells not only trigger the secretion of microparticles derived from platelets, but also stimulate those derived from tumor cells and those with both tumor and platelet markers. All of these microparticles recruit macrophages and activate their tumor‐killing activities, eventually causing tumor cell‐cycle arrest.^46^ Taken together, the role of platelets in tumor growth remains controversial and requires further investigation.

The platelet homing by tumors is selective. Chen et al.^47^ observed that the proliferative environment of pancreatic cancer can reject platelets, but is not related to a strong “homing” affinity. In addition, different effects mediated by activated platelets were observed in breast cancer and pancreatic cancer models.^47^ This suggests that the “homing” effect of platelets to different types of tumors may be highly correlated with the blood supply of tumors and influenced by the distinct microenvironments of various types of cancer. More than 90% of pancreatic cancers are composed of highly fibrotic collagen stroma, which greatly compress the microvessels in the tumor, leading to a significant decrease in effective blood perfusion.^48^ This indicates that the high expression of stroma can reduce blood perfusion and thus, affect the homing effect of platelets. Besides, as different tumor types have an impact on platelet homing ability,^47^ this limits the applicability of platelet‐based targeted therapeutic strategies for tumors.

### 3.2 Platelets and tumor cell metastasis

During tumor metastasis, individual tumor cells are unable to fight against the body's attack. The majority of tumor cells entering the bloodstream die within a short period of time under the action of the host immune system and the mechanical shear of the bloodstream. Only a very small number of tumor cells with high viability and metastatic potential interact with platelets and the coagulation system in the bloodstream can help form small cancer clots that escape immune clearance, thus metastasizing to distant sites.^49^ In previous experiments with mice, it was found that the metastasis of cancer cells was significantly reduced when the number of platelets was reduced or lost,^18^ suggesting that platelets may aid tumor cells to metastasize.

Anoikis is a special cell‐programmed death induced by cells breaking into contact with the extracellular matrix or adjacent cells. Overcoming the anoikis has a positive effect on tumor metastasis. Haemmerle et al.^50^ demonstrated that platelets induce resistance to anoikis in vitro, which is critical for metastasis in vivo. Platelets can contact directly with tumor cells or release some bioactive molecules to facilitate metastasis. In the blood vessels, platelets can prolong the time that tumor cells stay. In addition, P‐selectin on platelets can bind to tumor cells along with fibrous proteins, which can cause tumor cell‐induced platelet aggregation (TCIPA).^51^ The process can protect circulating tumor cells (CTCs) from clearance. In oral squamous cell carcinoma, podoplanin (PDPN) is a transmembrane glycoprotein on the surface of tumor cells. C‐type lectin‐like receptor‐2 (CLEC‐2) is located in the platelets which can combine with PDPN. The interaction between PDPN and CLEC‐2 can activate the platelets. The activated platelets can accumulate and enhance the invasion and metastasis of the tumor cells.^52^ It is known that chemical signals, such as NF‐κB and transforming growth factor‐β (TGF‐β) signals released by platelets, can make the tumor cells more invasive.^53^ In a recent research, the immune checkpoint molecule pair HLA‐E:CD94‐NKG2A mediated the interaction between CTCs and NK cells. RGS18 secreted from platelets improved the expression of HLA‐E via AKT‐GSK3β‐CREB signaling. RGS18 can facilitate the metastasis of tumor cells.^54^

However, another experiment showed that platelets inhibited the growth of liver cancer in mice of nonalcoholic fatty liver disease. The antitumor function of platelets is mediated by P2Y12‐dependent CD40L release, which leads to the activation of CD8 T cells by the CD40 receptor.^55^ Platelets can also inhibit tumor cell proliferation by blocking the cell cycle at the G0/G1 phase.^56^ The role of platelets in tumor progression is complex and diverse, requiring further study.

#### 3.2.1 Metastasis through immune escape

One of the ways in which cancer cells in the blood can eventually metastasize successfully is by allowing platelets to coat their surface, thus creating a barrier that makes the cancer cells invisible to cellular components of the immune system. Mechanistically, tumor cells enter the systemic circulation and release thrombin to rapidly recruit and activate platelets. By binding to P‐selectin, which is highly expressed on the surface of activated platelets, together with fibrin deposition,^57^ tumor cells rapidly form a microthrombotic barrier,^58^ a process known as TCIPA.^51^ TCIPA is a prerequisite for the survival and metastasis of tumor cells in the vascular system. It induces platelet activation and aggregation mainly through the secretion of various active substances. This forms a layer of platelets wrapping the tumor cells, thus hiding them from being detected by immune cells and also avoiding injury from the high‐speed shear stress of blood flow. In addition to protecting the integrity of CTCs, platelets can also transfer major histocompatibility complex class I molecules to the surface of TCIPA aggregates by secreting exosomes,^59^ which interferes with the recognition of circulating NK cells and helps CTCs evade immune surveillance. Platelets recruit monocytes and granulocytes to cancer cell blocking sites, which together shape a protumor environment conducive to metastasis,^14^ thus allowing CTCs to successfully cross the endothelium into the early metastatic niche and subsequently form metastatic foci. The most widely used antiplatelet coagulation drug in oncology treatment is aspirin. Aspirin can improve the prognosis of mice with chronic hepatitis B and reduce the incidence of hepatocellular carcinoma, and also inhibit the growth and metastasis of osteosarcoma through the NF‐κB signaling pathway. In recent years, numerous clinical studies have shown that antiplatelet therapy can reduce tumor metastasis and prolong patient survival.^60^ For instance, daily oral aspirin (≥75 mg/day) prevented distant tumor metastasis and reduced tumor morbidity and mortality and, furthermore, the survival of patients with CRC diagnosed with the PIK3CA variant can be prolonged by treating with routine oral aspirin (325 mg/day).^61^ In conclusion, platelets are necessary in tumor growth, angiogenesis, metastasis, and evasion of host immune response through TCIPA. In the comprehensive treatment of tumors, antiplatelet agents can be applied to inhibit platelet activation, contributing to the reduction tumor metastasis. Antiplatelet agents may be considered in combination with surgery, local therapy, and radiotherapy to increase their efficacy in treating tumors, but a large number of clinical studies are still needed to confirm this.

TGF‐β released from platelets suppresses the activity of antitumor T cells and is a cytokine that exerts an immunosuppressive effect, enhancing the growth and metastasis of cancer cells.^62^ Primarily synthesized as an inactive proprotein, TGF‐β requires GARP (glycoprotein‐A repetitions predominant), a docking receptor for latent TGF‐β, which can promote the furin‐dependent cleavage of TGF‐β proprotein, eventually leading to the TGF‐β maturation.^63^ Previous research indicated that GARP expressed in tumor cells induced the activation of latent TGF‐β, and hence, matured TGF‐β could upregulate the expression of Foxp3 which rendered effector T cells immune‐suppressive.^64^ This tolerogenic FoxP3/GARP/TGFβ axis, therefore, favors tumor immune evasion. TGF‐β suppresses the differentiation of cytotoxic T cells and increases the regulatory T cells which subsequently inhibit the activation of NK cells and T cells.^44^

During the interaction between platelets and NK cells, tumor cells also express normal platelet surface receptors, which prevent NK cells from recognizing tumor cells, leading to tumor immune escape and increasing the early survival rate of metastatic cancer cells.^65^ For instance, platelets express “a disintegrin and metalloproteinase” (ADAMs) on their surfaces, and one of the members called ADAM10 participates in the shedding of the stress‐induced NKG2D ligands such as MICA/B and ULBP2 on tumor cells.^66^, ^67^ PD‐1 and CTLA‐4 belonging to the immune checkpoint proteins can interact with TCIPA and Tregs to evade the immune system to achieve the goal of metastasis.^68^ In normal conditions, NK cells with NKG2D (an activating immunoreceptor) on their surfaces can recognize NKG2D ligands expressed on tumor cells, thus exerting cytotoxic effects.^69^ However, the cleavage of NKG2D ligands on tumor cells by ADAM10 damages this tumor‐lysis function.^69^ The reactivity of NK cells is modulated by activating inhibitory surface receptors, such as tumor necrosis factor (TNF) receptors.^70^ One of the TNF family members called glucocorticoid‐induced TNF‐related ligand is coexpressed with P‐selectin on platelets owing to platelet activation mediated by tumor cells.^71^ Although this molecule does not affect the activation or function of platelets, it contributes to platelets coating with tumor cells and binds to glucocorticoid‐induced TNF‐related protein on NK cells, reducing Interferon‐γ (IFN‐γ) generation and NK cell cytotoxicity.^71^ A study demonstrated that solid tumor cells induced platelets to upregulate the expression of the platelet‐derived receptor activator of NF‐κB ligand (RANKL), another member of the TNF family.^70^ Receptor activator of NF‐κB (RANK) is displayed on NK cells, which weaken the NK tumor‐killing effects.^64^ Individuals with solid tumors have NK cells with upregulation of RANK, which interact with RANKL, weakening the antitumor effects of NK cells, with a more prominent effect on IFN‐γ generation and a weaker effect on cytotoxicity.^64^ This impairs NK cell immunosurveillance and aids tumor evasion. Moreover, TGF‐β can directly influence NK cells through reducing NK cells’ activating receptors and dampening IFN‐γ transcription.^72^, ^73^ Platelet‐secreted TGF‐β can also downregulate the expression of NKG2D, an activating receptor on the surface of NK cells, resulting in a decrease in the antitumor activity of NK cells.^74^ Experiments in vivo showed that in thrombocytopenic mice, hematogenous metastasis of tumor cells was reduced. But when NK cells were precleared, hematogenous metastasis significantly increased.^63^ Therefore, through weakening NK cells and other immune components by TGF‐β, platelets foster tumor metastasis. Overall, platelets can affect the antitumor effects of NK cells.

In some cases, platelets also mediate immune escape through activating immune‐inhibitory receptors. For instance, in myeloproliferative neoplasms where the bone marrow generates an excessive amount of blood cells and platelets, oncogenic JAK2^V617F^ (Janus kinase 2) mutation leads to phosphorylation of STAT5 (signal transducer and activator of transcription 5) and STAT3, which upregulates the PD‐L1 expression in mutated cancer cells including platelets.^75^ This results in PD‐L1‐mediated immune escape via suppressing cell cycle progression and metabolism of T cells.^75^

#### 3.2.2 Tumor−platelet interaction promotes metastasis through angiogenesis

Continuous tumor proliferation requires an adequate blood supply from new blood vessels to carry nutrients, oxygen, and metabolites necessary for growth. In addition to providing nutrients and water for tumor growth, new blood vessels spread tumor cells to distant areas and form new metastases in different parts of the body. The TME and stromal cells are the main factors that promote neovascularization, and these vascular networks provide the environment for tumor development, progression, and metastasis.^76^

Tumor−platelet interaction promotes angiogenesis mainly through two ways. On the one hand, cancer cells usually secrete growth factors such as vascular endothelial growth factor (VEGF) to induce vascularization. Platelets can then absorb VEGF, lipids, protein hydrolases, and other components from the environment that are beneficial to vascular renewal, and then release them when being stimulated to promote vascular renewal.^38^ In breast cancer, platelets sequester proangiogenic factors from aggressive tumor tissues and then send them to nonaggressive ones, hence facilitating vascularization in the indolent tumors.^77^ Solid tumors can also express TF, which interacts with factor VII/VIIa, resulting in the formation of thrombin.^78^ On the other hand, vasculature inside tumors plays a crucial role. The overexpression of TF by tumor endothelial cell system (ECs) is associated with increased expression of VEGF‐A and microvessel density (MVD).^79^ Indeed, transfecting TF in cancer cell lines induced increased transcription of VEGF and reduced transcription of thrombospondin 1 (TSP1) and thrombospondin 2 (TSP2) secreted by platelets, indicating the proangiogenic role of TF.^70^ Both TSP1 and TSP2 are related to antiangiogenesis and are endogenous angiogenesis inhibitors with reduced or no expression in a variety of tumor tissues. Moreover, the expression of TSP1 and TSP2 was negatively correlated with MVD. Besides, aggressive tumor‐activated bone marrow cells can upregulate CD24 expression on indolent tumor cells and enhance the infiltration of VEGFR2^+^ tumor‐associated endothelial cells.^77^ This promotes the platelet‐cloaking of tumor cells and tumor vascularization.^77^

Platelets are the main carriers of the proangiogenic factors VEGF, platelet‐derived growth factor (PDGF), and basic fibroblast growth factor (β‐FGF). PMPs can also secrete VEGF, PDGF, FGF, and matrix metalloproteinase (MMPs) to promote tumor angiogenesis, increasing the chance of distant metastasis of tumor cells.^38^ VEGF is one of the most important angiogenic proteins transported and released by platelets.^80^ PDGF is necessary for the growth of smooth muscle cells, fibroblasts, and glial cells.^81^ Overexpression of PDGFs and platelet‐derived growth factor receptors (PDGFRs) is associated with human cancer and is significantly associated with malignant consequences.^82^ It shows that anti‐VEGF drug therapy can inhibit the signaling pathways of growth factors and thus targeting angiogenesis and distant metastasis.^41^ Through releasing α‐granules, platelets can also promote bone marrow‐derived cells to mobilize into the blood and facilitate their homing to tumor‐associated neovasculature in a VAMP‐8‐dependent way.^83^ Studies have shown that nucleolin hUTP14a upregulated the transcription and secretion of PDGF, thereby contributing to increased MVD in colon cancer tissues.^42^ Platelets may induce a “wound healing” response via degranulation of PDGF, CXCL12, and VEGF‐A. This subsequently recruits and stimulates CAF and myeloid cells, and leads to extracellular matrix (ECM) deposition, enhancing cancer angiogenesis.^84^

Interestingly, apart from proangiogenic factors, platelets are also the sources of antiangiogenic molecules, such as TSP1, endostatin, and platelet factor‐4.^85^ It indicates that simulators and inhibitors of angiogenesis are finely controlled and released through different α‐granules, depending on specific stimuli.^86^ For example, ligation of protease‐activated receptor PAR1 triggers the selective release of granules containing VEGF, whereas PAR4 activation induces the release of granules loaded with endostatin but not those with VEGF.^86^ However, an opposing opinion is raised that platelet secretion does not rely on the contents but is random, kinetically controlled, and is associated with agonist potency.^54^

The molecules expressed on the platelets may also participate in angiogenesis. For example, CD40 ligand (CD40L) is a molecule expressed on the surface of platelets and it can be cleaved to form soluble CD40L.^87^ This molecule stimulates the endothelial progenitor cells, increasing the secretion of MMP‐9 from these cells possibly via the p38 MAPK signaling pathway, which supports angiogenesis.^88^ Soluble CD40L also binds CD40 expressed on platelets, further activating platelets.^87^ Platelets can interact with host endothelial cells via GPIIb/IIIa, an integrin complex expressed on platelets, which plays a vital role in platelet‐mediated endothelial cell spouting.^89^ Vitronectin receptor αvβ3, which are expressed on endothelial cells, tumor cells, and platelets, may also play a role in the angiogenesis mediated by the interaction among these cells, since blockade of this molecule (merely on ECs or tumor cells) remarkably inhibits cancer cell invasion.^89^

Platelets are important in maintaining vascular integrity and hemostasis within tumors. Research has shown that induced thrombocytopenia causes hemorrhage in tumors, resulting in apoptosis of tumor cells and decreased cell proliferation.^90^ Further, thrombocytopenia‐triggered bleeding of tumors was dampened by resting platelets but not by degranulated ones, which indicated the granular contents of platelets play a fundamental role in preventing intratumor bleeding by platelets.^90^ For instance, some factors released by platelets, such as angiopoietin‐1, have the effect of maintaining microvascular integrity probably through counteracting with VEGF‐A secreted by tumor cells, which in turn reduces the invasion of immune cells into tumor tissue and contributes to tumor metastasis.^91^ Besides, another study also suggested that intratumor hemorrhage can be induced by innate immune cells so platelets may hamper local tissue damage via regulating leukocyte recruitment, thus maintaining the integrity of blood vessels.^92^ What is more, recent research showed that platelet‐derived growth factor B (PDGFB) promoted and maintained vascular integrity in the TME by promoting pericyte recruitment.^93^

#### 3.2.3 Metastasis through EMT

Platelets play an important role in the adhesion and extravasation of tumor cells to surrounding tissues and organs. By releasing cytokines, platelets induce the tumor cells to undergo EMT. Tumor cells with altered phenotype cross the basement membrane and vascular endothelium, transferring to the target organs. The activated blood cells simultaneously release ATP and MMPs, which induce the opening of the vascular endothelial barrier and degrade the vascular basement membrane structure, prompting the tumor cells to infiltrate the blood vessels and invade the surrounding environment.^94^

**FIGURE 3:**
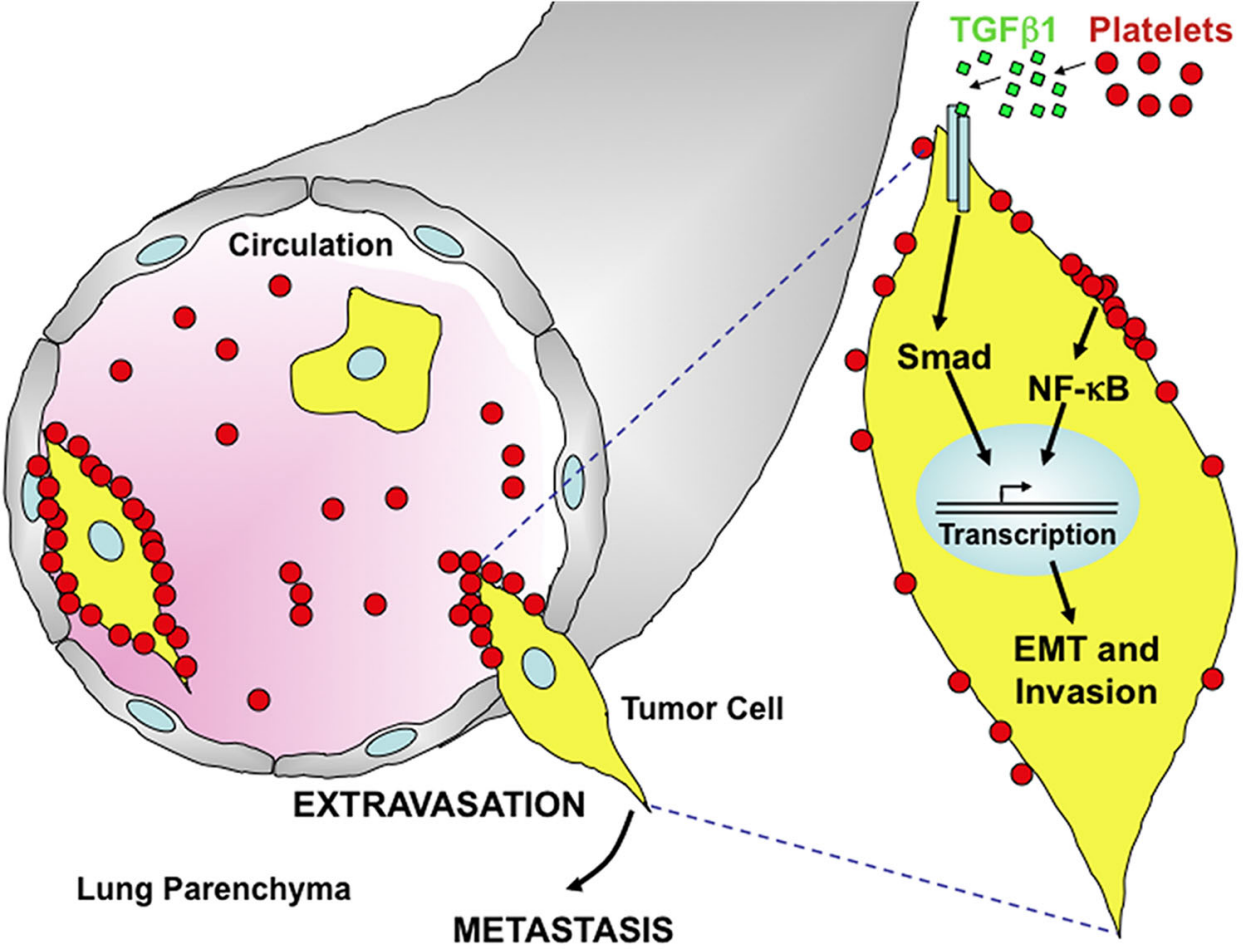
Platelets interact with tumor cells to facilitate EMT‐like transition. Platelets secrete TGFβ−1, which can activate signaling pathway in the tumor cells. Reproduced with permission from reference 95.

PDGF is a basic protein that is isolated from human platelets and stored in platelet alpha granules.^95^ In addition, PDGF can be synthesized and released by vascular smooth muscle cells, fibroblasts, macrophages, and so on. PDGF released from tumor cells can induce proliferation and migration of vascular endothelial cells, smooth muscle cells, and tumor cells, and inhibit their apoptosis, thus playing a direct and indirect role in tumor angiogenesis.^96^ The role of PDGFs in tumor EMT was indicated in multiple studies.^97^ For instance, through direct contact with tumor cells or secreting TGF‐β, platelets activate the TGF‐β/Smad and NF‐κB signaling pathways to induce EMT of colon cancer cells (Figure 3),^98^ enhancing the invasive ability of tumor cells. Colon cancer cells treated with TGF‐β can express PDGF‐B, which is phosphorylated by platelets to cause EMT of tumor cells, but the ability of tumor cells to undergo distant metastasis is significantly reduced when the TGF‐β gene is knocked out.^99^ PDGF produced by highly activated platelets can also increase MMP2/9 expression and stimulate the p38/MAPK pathway to trigger EMT in platelet‐stimulated cholangiocarcinoma.^100^ This leads to tumor dissemination and metastasis.^100^ Upregulation of PDGF‐D in endometrial cancer cells can lead to tumor metastasis via promoting MMP2/9 expression and EMT.^101^ Direct contact of platelets with gastric cancer cells also contributed to the upregulation of EMT‐related genes, such as MMP9, leading to tumor migration.^102^

**FIGURE 4:**
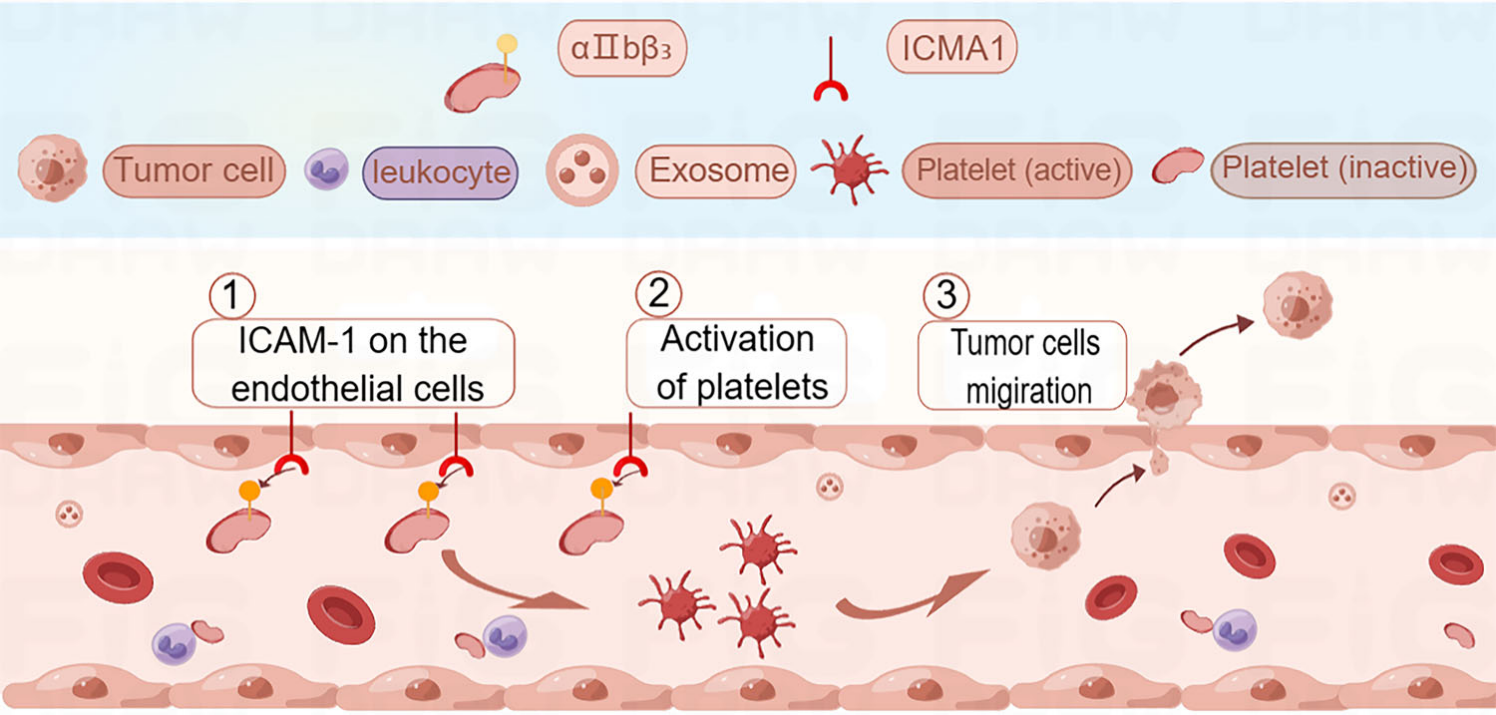
In the blood vessels, ICAM‐1 (intercellular adhesion molecule 1) expression on endothelial cells can activate the platelets, turning the inactive form into the active form. The activated platelets can foster the migration of cancer cells to form secondary tumors. In addition, platelets can interact with leukocytes. The figure is made using Figdraw.

The interaction between platelets and endothelial cells plays an important role in promoting the EMT of cancer cells. For instance, platelet priming of tumor cells significantly increased colon cancer adhesion to endothelium and the crosstalk also induced EMT, which was indicated by upregulation of snail 1 and β‐catenin and downregulation of E‐cadherin in a time‐dependent way.^46^ This eventually resulted in more metastatic sites in other organs.^46^ In addition, microparticles derived from platelets or tumors or both may also prime the endothelial cells through increasing intercellular adhesion molecule 1 expression on ECs, thus fostering the adhesion of cancer cells at metastatic sites (Figure 4).^46^ Besides, after platelets are activated, P‐selection stored in the platelet granules can be transferred to the platelet surface and then bound to P‐selectin ligands expressed on the tumor cells.^103^ Increased P‐selectin expression enhances the ability of tumor cells to adhere to the vascular endothelium and contributes to tumor cell exudation.

Tumor cells are often characterized by expression of tumor‐specific antigens or overexpression of tumor‐associated antigens. These molecules may be involved in platelet‐induced tumor EMT. For instance, COX‐2 expression is upregulated in a variety of tumor cells, and its downstream metabolite TXA2 binds to the G‐protein‐coupled receptors P2Y and TBXA2R on the surface of platelets, inducing platelet activation to bind to tumor cells and promoting their hematogenous metastasis.^104^ Ward et al.^105^ demonstrated that the tumor‐associated antigen CD97 induces platelet activation and participates in the RHO‐GTP signaling pathway mediated by LPA, which induces EMT in tumors and improves the survival of tumor cells. Adams and Pang et al.^106^, ^107^ demonstrated that tumor cells can also express thrombin, and when tumor cells were coincubated with platelets activated by thrombin and transfused back to mice, the metastatic capacity of tumor cells was significantly increased.

Recent research showed that platelets can trigger tumor biosynthesis of 12S‐hydroxyeicosatetraenoic acid (12S‐HETE) and its esterification to plasma membrane phospholipids through tumor cell uptake of platelet‐derived extracellular vesicles expressing 12‐LoX. This may affect the expression of EMT marker genes and foster cancer metastasis.^108^ TBK1, which is the abbreviation of TRAF (tumor necrosis factor receptor‐associated factor) family member‐associated NK‐κB activator (TANK)‐binding kinase 1, was found as a novel mediator of platelet‐induced NF‐κB signaling through activating NK‐κB subunit p65.^109^ Thus, TBK1 contributed to EMT of mammary carcinoma cells, their invasiveness, and metastasis.^109^

## 4 ASSOCIATION BETWEEN PC AND CANCER

More than a hundred years ago, scientists have observed that there seems to be a correlation between platelets and cancer, and many cancer patients have increased PCs by many times or even up to 20 times. Moreover, the higher the PC, the more severe the disease and the shorter the survival time of cancer patients. However, it is only recently that scientists are slowly understanding what is behind the cause.

The change of PC can be used as a reference index for tumor occurrence, development, curative effect, and prognosis. The blood of patients with advanced stage is in a state of hypercoagulation, which may be conducive to tumor growth and metastasis.^110^ Increased PCs have been revealed to be a predictor of cancer in patients with occult malignancies^111^ and are consistently associated with progression‐free and/or worse overall survival in ovarian,^112^, ^113^ colorectal,^114^ lung cancer,^115^ gastric cancer,^103^ and breast cancer.^116^ Platelets are involved in tumor angiogenesis and cancer progression. High PCs in lung, colon, stomach, kidney, and gynecologic cancers are associated with shorter disease survival.^103^, ^108^, ^109^, ^110^, ^111^, ^112^, ^113^, ^114^, ^115^, ^116^, ^117^, ^118^, ^119^ Previously, scientists at the University of Exeter Medical School in the United Kingdom analyzed the records of 40,000 patients and found that more than 11% of men and 6% of women over the age of 40 with thrombocytosis were diagnosed with cancer within 1 year.^120^ In addition, for patients with two elevated PCs within 6 months, the chance of cancer diagnosis increased to 18% for men and 10% for women.^120^ In 2022, a nested case−control study, which involved more than 8 million subjects, reported that individuals with high PCs would have a diagnosis of cancer within 10 years after the blood test.^118^ These cancers include colon, ovarian, stomach, and lung cancers and the extent of the correlation between the diagnosis and high PC varies among them.^118^ In addition, PCs increased as these cancers approached the date of diagnosis.^121^ Platelets influence disease burden and treatment outcome in cancer patients and are involved in steps, such as cancer metastasis. Platelets also play an important role in protecting cancer cells from chemotherapy‐induced apoptosis and in maintaining the integrity of the tumor vascular system.^122^ In vitro tests have demonstrated that platelets increase resistance to 5‐fluorouracil and paclitaxel in colon and ovarian cancer cell lines.^123^ Lower PCs strongly increase the sensitivity to paclitaxel in mouse models of breast or lung cancer.^123^ This special function of tumor‐associated platelets (TAPs) provides a guarantee for tumors to maintain their rapid growth characteristics, so that tumor tissues will not be necrotic due to insufficient blood and nutrient supply.

**FIGURE 5:**
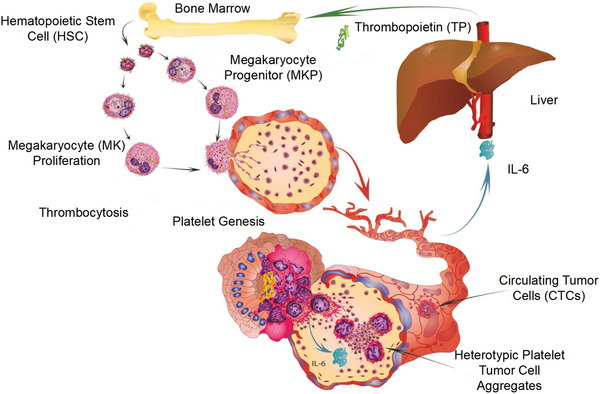
Thrombopoietin from the liver can enhance the production of platelets. In addition, IL‐6 from the tumor cells can stimulate the production of thrombopoietin, which can form a cycle. Reproduced with permission from reference 37.

The underlying mechanism of cancer‐induced increase in PC has been revealed. Cancer cells secrete various cytokines which may promote platelet formation. For instance, several types of tumor cells can secrete interleukin‐6 (IL‐6),^124^ which stimulates liver cells to produce more TPO, which in turn promotes the maturation of platelet‐producing megakaryocytes, leading to higher PCs (Figure 5).^125^ Besides, tumor cells can produce proteases, such as thrombin, cathepsin B, MMP‐2, and MMP‐14. These proteases may stimulate protease‐activated receptors (PARs), which mediate platelet activation. Specifically, through binding to PAR1, thrombin caused exposure of anionic phospholipids which facilitated platelet activation and blood clotting.^126^ Thrombin can also activate PAR4 but the response was restricted to platelet aggregation, akin to the response of platelets triggered by epinephrine, adenosine diphosphate (ADP), or other weak agonists.^127^

## 5 CHARACTERISTICS OF THE INFLAMMATORY MICROENVIRONMENT MEDIATED BY PLATELETS INTERACTING WITH LEUKOCYTES AND STROMAL CELLS

Mutations in normal cells are necessary for tumorigenesis, but mutations alone are not enough. Inflammation plays an important role in initiating tumorigenesis by destroying tissues, and leukocytes are a key part of this process.^127^ Study showed that platelet and leukocyte interactions mediate a range of inflammatory physiological and pathological processes.^128^ Under physiological conditions, resting endothelial cells keep platelets in a resting state. However, during endothelial injury, proteins of the subendothelial matrix are exposed to the lumen and can activate platelets. Activated platelets can recruit more platelets to aggregate and seal the wound on the one hand; on the other hand, they interact with leukocytes, causing leukocyte recruitment, activation, extravasation, phenotypic switching, and a series of alterations in functions, further enhancing the degree of tumor inflammatory environment, including immunosuppression.^129^

In the TME, platelet surface receptors and released active substances enhance platelet activation, forming TCIPA, which contributes to cancer thrombus formation and helps tumor cells escape immune attack and metastasis. Glycoprotein IIb‐IIIa is the platelet surface transmembrane receptor most involved in TCIPA. As early as 1987, it was observed that GPIIb/IIIa contributed to cancer thrombus formation during platelet aggregation (TCIPA) induced by lung cancer cells and glioma cells, helping tumor cells to escape the immune attack and thus tumor metastasis.^130^ Amirkhosravi et al.^131^ showed that oral or intravenous administration of XV454, an antagonist of GPIIb‐IIIa, to mice with lung metastases significantly reduced the binding of platelets to tumor cells and the occurrence of hematogenous metastasis of tumor cells. Cancer thrombus formation can block normal blood flow and the endothelial cells are activated, which promotes inflammation. Specifically, some receptors on endothelial cells will allow leukocytes, for example, neutrophils, to adhere to endothelial cells, migrate through blood vessel walls, and enter the tissues which trigger inflammation in distant organs in patients with cancer.^132^ Platelets can also be activated, releasing a large number of proinflammatory factors that regulate the TME. This drives the recruitment of leukocytes, attracting more leukocytes to the site, thus inflammation is exacerbated. This process also contributes to the remodeling of the extracellular matrix and angiogenesis. Apart from releasing factors to foster inflammation, activated platelets also express receptors on their surfaces that can interact directly with immune cells, such as p‐selectin and CD40L.^133^, ^134^

It is shown that knockout of Ral GTPases, a modulator of cancer metastasis in platelets, remarkably reduces the platelet surface expression of P‐selectin, which subsequently leads to a substantial decrease in platelet−leukocyte interactions.^135^ Thromboxane A2 (TXA2) is another substance derived from platelets that participate in controlling tumor metastasis.^136^ Specifically, as a prostanoid product of cyclooxygenase‐1, TXA‐2 favors the formation of intravascular premetastatic niches and the recruitment of monocytes or macrophages that foster metastasis.^136^ In addition, Zhang et al.^137^ discovered that neutrophil‐lymphocyte ratio (NLR) and platelet‐lymphocyte ratio (PLR) were strongly associated with cancer metastasis to lymph nodes. NLR also served as an independent prognostic factor for the overall survival of patients with gastric cancer.^137^

Platelets have recently been found to be involved in the formation of neutrophil extracellular traps (NETs),^138^ which are web‐like structures composed of DNA and antimicrobial peptides from neutrophils.^139^ On the front lines of the host defense response, the antimicrobial function of neutrophils has been adapted to fight infection and injury from different sources and degrees. In the past, the formation of NETs was thought to be an innate immune defense mechanism against severe bacterial infections.^140^ Recent studies have found NETs in biopsies of human tumors.^141^ NETs also stimulate the migration and invasion of cancer cells in vitro.^141^ Ren et al.^142^ demonstrated that surgical removement of tumors may cause stress that activated platelets via TLR4/ERK5/integrin GPIIb/IIIa signaling, fostering the aggregation of platelets with CTCs. These aggregates were then trapped by NETs which help the tumor cells to metastasize to distal sites.^142^ Thus, by disrupting the cross‐talk between NETs and platelets, cancer metastasis may be prevented after resection.^142^ Apart from cancer metastasis, NETs were also shown to induce procoagulant activity in patients with CRC.^143^ It was found that CRC patients had neutrophils that were more prone to produce NETs whose level also elevated with cancer progression.^143^ Subsequently, NETs promoted the exposure of phosphatidylserine on platelets and also induced the switch of endothelial cells to procoagulant phenotype.^143^ These led to the increased risk of thrombosis in cancer patients.^143^ In fact, there is a positive feedback loop between platelets and NETs. Specifically, NETs stimulate platelets via releasing substances, such as TFs, a platelet activator, from neutrophils.^144^ In turn, activated platelets also facilitate the formation of NETs.^145^

Generally, both neutrophils and platelets contribute to the construction of the TME. Patients with cancer tend to show an increase in the number of circulating neutrophils.^146^ The number of neutrophils is usually small but these cells remain key regulators of tumor development and progression.^147^ According to different properties and effects on tumor progression, neutrophils were divided into N1‐ and N2‐neutrophils.^148^ N1 neutrophils have an immunoactivating ability and tumor cell cytotoxicity, while N2 neutrophils secrete reactive oxygen species (ROS), arginase and peroxidase, and other molecules, which inhibit T cell and NK cell function and promote tumor.^149^, ^150^ The switch from antitumor N1 neutrophils to protumor N2 neutrophils can be induced by TGF‐β which is mainly derived from platelets.^151^

Neutrophils show a unique ability at each stage of cancer: from tumor initiation to primary tumor growth to metastasis.^152^ Nevertheless, during these processes, neutrophils have different phenotypes and sometimes even show opposite functions, indicating their plasticity in the TME.^153^ In addition, it is shown that platelets can form neutrophil‐platelet aggregates (NPAs) with neutrophils. The complex can boost the functions of neutrophils in the process of inflammation, which can worsen varieties of inflammatory diseases.^154^ However, it was unclear if NPAs could play a similar role in the TME. According to recent studies, it is known that the NLR and PLR may have a role in predicting tumor response and prognosis.^155^, ^156^ Maybe the two markers can reveal a completely new approach for the treatment of cancer patients, which is required to be explored further.

In addition, in the TME, platelets interact with the endothelial cells to help the initiation, metastasis, and angiogenesis to facilitate the progression of tumors. It was discovered that by secreting granule contents, such as 5‐hydroxytryptamine, platelet factor IV, TGF‐β, or directly adhering to damaged blood vessels, TAPs are able to maintain the integrity of tumor vascular endothelium and prevent intratumor hemorrhage.^157^, ^158^ The significant finding can provide nutrients for blood vessels.

CAFs, as a major component in the TME, exert essential effects on the development of tumors. A number of evidence suggested that only CAFs cannot affect the tumor. The interaction between other cells and CAFs can facilitate the growth of tumors. CAFs can be activated by PDGF signaling mediated by platelets. Activated CAFs can secrete some factors to affect the status of platelets.^159^ PDGFB released from platelets can enhance the accumulation of CAFs and deposition of extracellular matrix.^160^ Abundant studies indicated that PDGF is indispensable for the function of the CAFs. In addition, TGF‐β secreted by platelets can improve the proliferative ability of fibroblasts, which enhance the malignancy of tumors.^161^

## 6 PLATELETS AND CANCER TREATMENT

Platelet has been proven to be a biomarker for cancer prediction, detection, and prognosis in the future due to changes in its number, nucleic acid information, molecular expression, and transcriptional profile. Based on its interaction with tumors, scientists can perform anticancer treatments by disrupting their communication as well as using nanotechnology.

### 6.1 Platelets as a biomarker

The use of blood components as biomarkers for cancer prediction and detection is a promising development. In addition to CTCs and cell‐free DNA, cancer‐associated platelets are also promising biomarkers.^162^, ^163^, ^164^, ^165^ As platelets are the second most numerous blood cell in the blood, they can be isolated and counted easily. In addition, platelets also store various biomolecules released by cancer cells because they actively absorb and store proteins and nucleic acids from outside. Information on PCs, protein and mRNA profiles, and their activation status can be determined to monitor the presence and, to some extent, the location of tumors. However, as platelets are prone to contamination by white blood cells during isolation and platelet activation during isolation can affect the results of the analysis, establishing a stable methodological procedure for platelet purification would help to make platelets an official cancer biomarker.

PC was shown to be an independent prognostic factor predicting the survival of patients with breast cancer.^166^ It was negatively correlated with overall survival and disease‐free survival in those patients.^166^ Another research indicated that PC also had prognostic value in operable, nonmetastatic breast carcinoma and the preoperative PC can be combined with the level of CTCs to predict cancer progression.^167^ A novel study indicated that platelet RNA may be a liquid biopsy biomarker for pan‐cancer at an early stage. This may compensate for the shortage of mutated cf DNA because its plasma level is very low during the early cancer stage.^168^ Besides, nuclear receptor‐interacting protein 1 RNA, a specific circular RNA of platelets, can also be used for detecting non‐small cell lung cancer (NSCLC) due to its differential expression between NSCLC patients and healthy individuals.^169^ When researchers decreased the number of platelets, the ability of chemotherapeutic drugs to poison cancer cells increased, indicating that platelets have an antichemotherapeutic drug effect.^123^, ^170^ Studies also found that cancer patients with higher PCs had poorer postchemotherapy outcomes and less effective treatment.^171^ These findings seem to suggest that inhibiting the number or activity of platelets may improve the efficacy of chemotherapy drugs. For instance, it has been shown that the combination of multi‐kinase inhibitor sorafenib with resminostat (an inhibitor of histone deacetylase) significantly impeded platelet‐triggered invasion of hepatocellular carcinoma, with downregulated platelet‐induced CD44 expression (a cancer stem cell marker) and EMT genes.^172^ Additionally, the efficacy of this combination treatment depends on PC.^172^

**FIGURE 6:**
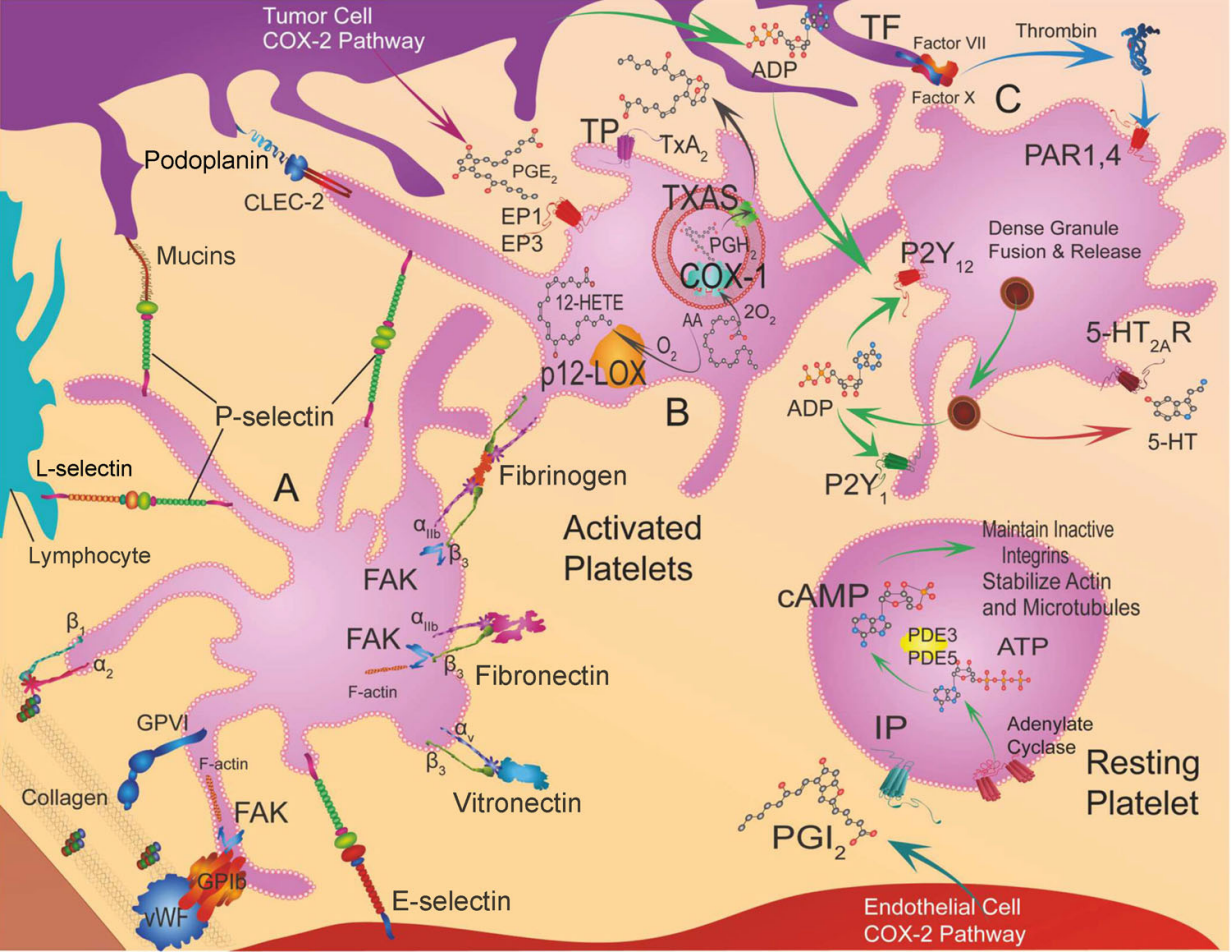
Presentation of platelet receptors and intracellular signaling among several kinds of cells. In platelet A, it shows that adhesion molecules on the platelets which can interact with endothelial cells, lymphocytes, other platelets. In platelet B, TXAS can generate TxA2, which can activate TP receptors. And cyclooxygenase‐1 (COX‐1) can synthesize PGH2, which can stimulate EP1 and EP3 receptors. In platelet C, the action of protease‐activated receptors (PAR1 and 4) can stimulate the platelets. Tumor cells can release ADP to activate P2Y1 or P2Y12 receptors. These targets provide possible therapies. Reproduced with permission from reference 37.

Apart from nucleic acids and PC, molecules expressed on platelets have also been used to reflect cancer progression (Figure 6). For instance, in NSCLCs, PD‐L1 can be transferred from tumor cells to platelets and the PD‐L1 level of platelets can reflect the total expression of this molecule on tumor cells.^173^ Thus, detecting platelet PD‐L1 level overcomes the difficulty in histological quantification of PD‐L1 level in tumor biopsies, and it can also predict the response of cancer cells to immune‐checkpoint inhibitor therapy.^173^ Substances secreted by platelets may also reflect tumor development. For instance, there is a positive correlation between the levels of PDGFR and tumorigenesis. Thus, a novel biosensor for monitoring cancers was designed to detect the PDGFR based on a charge‐based affinity bait molecule, which was superior to using antigen‐antibody reactions for detection.^174^ In glioblastoma (GMB), circulating platelets often express or produce various proangiogenic factors, such as P‐selectin and VEGFRs, differentiating patients with GME from healthy individuals. Thus, by measuring the expression level of these cargoes of platelets, the development of GBM may be monitored.^175^ Platelets also produce microparticles which can be combined with neutrophil‐derived microparticles and neutrophil/lymphocyte ratio to construct a predictive model for NSCLC development.^176^

The transcriptional profile of normal platelets differs from that of platelets isolated from patients with platelet‐associated diseases.^177^ Based on this evidence, platelets are an attractive platform for cancer diagnosis and prognosis. Specifically, scientists have focused on tumor‐educated blood platelets (TEPs) as these platelets changed their transcription profile during the cross‐talk with tumor cells.^178^ It has been reported that through sequencing mRNA data of TEPs, multiple cancers can be diagnosed with accuracy reaching 90%.^178^ Besides, this RNA information can also be used to identify locations of primary tumors and specific tumor mutations, such as KRAS and PIK3CA mutations.^178^ Further, diagnostic and prognostic readout of TEP RNA sequencing can be optimized by swarm intelligence, which leads to accurate detection of early and late‐stage NSCLC and early CRC.^179^, ^180^ The expression of TEPs mRNAs is different between healthy individuals and cancer patients. For example, Xing et al. discovered that the integrin alpha 2b (ITGA2B) RNA level of TEPs can be used as a candidate marker for differentiating malignant lung nodules from benign ones, and diagnosing stage I NSCLC.^182^

With the progress of the molecular biology of tumors, the study of platelet‐based RNA sequencing and its biological indicators will potentially provide a series of convenient and rapid molecular biology test markers for early diagnosis, prognosis determination, and follow‐up of clinical tumors (Table 1). It can provide a valuable platform where the diagnosis and prognosis can make progress.

**TABLE 1:** Biomarkers of platelets in cancer diagnosis and monitoring.

Biomarkers	Correlation with platelets	Application	Reference
Platelet counts	Intrinsic characteristics	Nonmetastatic breast carcinoma/drug response	^123^,^163^, ^170^, ^171^
NRIP1 RNA, circRNA	The differential expression of RNA in platelets between healthy people and cancer patients	Pan‐cancer/NSCLC	^168^, ^169^
PD‐L1	PD‐L1 can be transferred from tumor cells to platelets; PD‐L1 levels of platelets can reveal the therapeutic effects	NSCLCs/immune‐checkpoint inhibitor therapies	^173^
PDGFR	Platelet‐derived growth factor is a proangiogenic factor isolated from human platelets, platelet‐derived growth factor receptor is a member of the caseomic acid protein kinase family, which can promote the chemotaxis, division and proliferation of cells in the body, growth and development, wound repair	Monitoring	^174^
VEGFRs	Platelets express or produce various proangiogenic factors like VEGFRs	GMB	^175^
Microparticles	Produced by platelets to detect the progression of cancer	NSCLC development	^176^
mRNA profiles	The expression mRNA of platelets between healthy people and cancer patients	Diagnosis/identification, location, malignancy, stage, and mutation of tumors	^177^, ^178^, ^179^, ^180^, ^181^

Abbreviations: circRNA, circular RNA; NRIP1, nuclear receptor‐interacting protein 1; PDGFR, platelet‐derived growth factor receptor; PD‐L1, programmed death ligand 1; VEGFR, vascular endothelial growth factor receptor.

### 6.2 Targeted therapy strategies based on platelet−tumor interactions

The close interaction between platelets and tumors makes the use of platelets as a drug delivery vehicle or platform extremely attractive. Therefore, platelet‐based targeted therapeutic strategies have been attracting the attention of researchers and are increasingly being applied in oncology treatment.

#### 6.2.1 Disruption of the crosstalk between platelets and cancers

Due to the close relationship between platelets and cancer cells, platelets can serve as a target for antitumor treatments.

As most tumor cells require abundant blood supply and reside in highly vascularized environment, antiangiogenic treatment can be effective against cancer development. However, this treatment has drawbacks and may not improve prognosis.^182^ Fortunately, focal adhesion kinase (FAK) in platelets was demonstrated to be a promising target, which modulated the platelet extravasation into TME, further inducing the platelet−tumor interaction.^183^ It has been shown that after cessation of antiangiogenic therapy, the augmented infiltration of platelets in the tumor was associated with cancer rebound which can be strongly inhibited by FAK inhibitors or FAK‐deficient platelets.^183^ Moreover, a combination of FAK inhibitors and antiangiogenic drugs, such as anti‐VEGF antibody bevacizumab, has strong effects in killing tumor and can prevent the negative effects caused by the withdrawal of antiangiogenic drugs.^183^

In vitro studies showed the inhibition of specific molecules released by tumor cells can provide treatment. Human colon adenocarcinoma caco‐2 cell line released ADP and MMP‐2 to induce platelet activation and aggregation.^174^ When ADP and MMP‐2 are inhibited, respectively, the binding of tumor cells and platelets is reduced.^187^ Therefore, when lack of these substances, the expression of platelet GpIIb/IIIa, GpIb, and P‐selectin can be downregulated. Another study indicated that α‐Hederin suppressed the platelet‐activating factor receptor (PTAFR), thus blocking the PAF/PTAFR pathway that normally induces tumor cell migration and invasion.^185^ In addition, the expression of MMP‐2 can also be hampered by α‐Hederin via preventing the activation of STAT3 in platelet‐activating factor (PAF)‐stimulated tumor cells.^170^ Therefore, by targeting platelet activation, tumor development can be hindered. The interaction between CLEC2−PDPN exerts significant effects on metastasis, which can be an underlying target in tumor therapy. By blocking the interaction, the invasion and metastasis can be prevented.^123^

In recent decades, PDGFR has become a popular target in treating cancers. For instance, it has been shown that in osteosarcoma, specific inhibition of PDGFRα/β kinases by pyrimidine‐2,4‐diamine derivatives can significantly kill tumor cell lines.^186^ Another study demonstrated that PDGFRC was overexpressed in triple‐negative breast cancer patients and was associated with the patients’ survival.^187^ Additionally, after PDGFC and its receptors are inhibited, the drug effect of doxorubicin (DOX) can be enhanced, with more tumor apoptosis than only using DOX.^187^ Nayeem et al. discovered that PDGFRα was overexpressed in prostate cancer and the phosphorylation of this molecule and its downstream effector such as Akt was associated with tumorigenesis. However, the PDGFRα/Akt axis can be inhibited by Imatinib, a tyrosine kinase inhibitor, suppressing the growth and metastasis of tumor cells.^188^ In the therapy of hepatocellular carcinoma, JI‐MT, a novel indomethacin derivative, showed cytotoxic effect in the cancer cell line and reduced the liver cancer nodules in patients, via inhibiting PDGFR‐α.^189^ The overexpression of PDGFR‐α has also been found in GMB multiforme and HIF1α inhibitor echinomycin can target HIF1α‐PDGFD/PDGFRα‐AKT pathway, promoting the apoptosis of tumor cells at normoxia or mild‐hypoxia.^190^

Besides, as platelets can also assist tumors to evade the immune system, a related therapeutic strategy can be designed. For instance, a study indicated that platelets can increase the expression of PD‐L1 on lung and renal cancer cells, which resulted in tumor immune invasion.^191^ However, this can be reversed by eptifibatide, an antiplatelet drug. This suggests that antiplatelet drugs may be used as adjuvant to immune checkpoint inhibitors to treat tumors.^191^ The chimera antigen receptor (CAR) T cell therapy changed the synthesis of PAFs, which can be indicated by altered levels of lysophosphatidylcholines, a substance involved in the PAF synthesis, in multiple myeloma patients treated with CAR‐T.^170^ This suggests that treatment of multiple myeloma by CAR‐T therapy may be promoted by targeting PAF remodeling.^170^ The characteristics of platelets can provide directions for targeted therapies. Platelets can be involved in the process of coagulation. When blood vessels are damaged, TFs are released to initiate coagulation. During the process, platelets can accumulate. If the damage is on the tumor blood vessels, platelets with tumor‐killing drugs can also reach the damaged sites. These platelets were modified to target the tumor cells instead of normal cells. Immune checkpoint inhibitors can target the tumor cells by activating T cells.^68^ It is known that immune checkpoint HLA‐E:CD94‐NKG2A can help the tumor cells to evade the immune system. By targeting the immune checkpoint, it can provide new clews for the targeted therapy.^69^

#### 6.2.2 Application of nano‐modified platelet carriers in anticancer therapy

In recent decades, nanotechnology has thrived and an increasing number of nanoparticles have been combined with platelets to achieve better therapy against cancers.

PMs have various molecules that can interact with cancer cells. Therefore, nanoparticles decorated with molecules derived from platelets have been explored. A recent study designed a biomimetic nanoparticle coated with PM, which contained resiquimod (R848), an agonist of toll‐like receptor, and can locally deliver the drug intratumorally.^192^ Due to the biocompatibility of the PM, this R848‐loaded nanoparticle can stay in the tumor environment for a longer time with a higher affinity for cancer cells, which results in prolonged and enhanced antitumor immunity.^192^ Likewise, PM‐based nanoparticles can also be loaded with apatinib, an antiangiogenic drug, and vascular disruption agents, which can amass in tumor tissues owing to adhesion molecules on the membrane attaching to impaired vessels. This can effectively damage vessels and disrupt tumor angiogenesis.^193^ PM has also been applied to novel cancer treatment, such as glucose oxidase (GOx)‐based starvation therapy.^194^ Specifically, GOx‐functionalized PMs have been made to coat manganese dioxide which is a nanoshell assembling DOX‐loaded nanoparticles. This results in enhanced and more targeted tumor‐killing effects.^194^ Li et al.^195^ discovered that compared with free DOX, DOX attached to nanodiamonds coated with PM showed a longer time staying in blood circulation and stronger inhibition of tumor growth. PM has been used to camouflage single‐atom nanozyme. For instance, mesoporous Fe single‐atom nanozyme coated with platelets has been made, which can destroy mitochondria via hampering the expression of heat shock proteins and thus strengthen the mild‐temperature photothermal therapy.^196^ Interestingly, scientists have made a membrane combined with membranes of both platelets and tumor cells. This hybrid membrane was used to coat the β‐mangostin‐loaded nanoparticles, showing an improved ability targeting glioma.^197^ Taken together, due to the coating by PM, drug‐loaded nanoparticles tend to accumulate in tumor sites and have stronger therapeutic potency, better biocompatibility, and less systemic side effects on essential organs.

Apart from PM, platelet vesicles have also been investigated in cancer treatment combined with nanotechnology. For example, platelet exosomes were used, together with photothermal sensitive liposomes, to incorporate GOx and ferric ammonium, thus constructing a nanosystem controlled by a laser (FG@PEL).^198^ Photothermal effect can induce vascular damage, which activates platelet exosomes that often stick to tumor cells due to their biological property.^198^ This allows FG@PEL to exert its cascade targeting effect against tumor.^198^ For the treatment of pancreatic cancer, platelet vesicles were used to coat RSL‐3, a drug that can pass through the stroma and disrupt tumor angiogenesis. Due to platelet vesicles, the uptake of the drug by tumor cells increased, which enhanced tumor embolism and cell death.^199^

The capture of platelets by P‐selectin was also explored. For example, nanoparticles coated with a P‐selectin‐targeting peptide (PSN peptide) were constructed, and the core of these nanoparticles was filled with ticagrelor or celecoxib.^200^ Through capturing activated platelets, these PSN peptide‐modified nanocarriers have improved capability in accumulating at the primary tumor and premetastatic sites, hence robustly inhibiting platelet−tumor interaction and inflammation.^200^ As TAPs are often captured by primary tumor cells to facilitate the development of premetastatic niches, these nanoparticles also inhibit tumor metastasis.^200^

Without combination with nanotechnology, platelets themselves can act as carriers to deliver drugs. For instance, Bhandarkar et al.^201^ designed a quercetin (QCT)‐loaded platelet which had high encapsulation efficiency due to the canalicular system in platelets that allowed the drug to be loaded into the cytoplasm of platelets. These QCT‐loaded platelets showed great ability to kill GMB cells in vitro and are promising to target GMB tumor in humans.^201^ Also, platelets can be loaded with gold nanorods (AuNRs) carrying photothermal agents.^202^ These AuNR‐loaded platelets can stay stable in the blood and target tumor cells precisely after laser irradiation.^202^ Therefore, platelet inhibitors play an essential role in tumor therapy (Table 2).

**TABLE 2:** Summary of the application of platelet inhibitors in tumor therapy.

Platelet inhibitors	Detailed introduction	Mechanism	Reference
FAK inhibitors	FAK in platelets was demonstrated to be a promising target, further causing the platelet−tumor interaction	Controlling the platelet extravasation	^183^
ADP/MMP‐2 inhibitors	ADP and MMP‐2 released from tumor cells	Reducing the activation and aggregation of platelets	^184^
PTAFR inhibitors	The receptor of platelet‐activating factor (PAF). PAF can activate the platelets	Blocking PAF/PTAFR pathway	^185^
α‐Hederin	Expression of matrix metalloproteinase‐2 can be hampered by α‐Hederin	Inhibiting the activation of STAT3	^185^
PDGFR inhibitors	PDGFR expressed in the tumor cells	Inhibiting PDGFRα/β kinases	^186^, ^187^
Imatinib	Inhibit PDGFRα/Akt and disrupting the action of PDGF	Tyrosine kinase inhibitor	^188^
HIF1α	Inhibiting the expression of PDGFR in tumor cells	Inhibitor echinomycin	^190^
Eptifibatide	PD‐L1 inhibitor	Immune checkpoint inhibitors	^191^
CAR‐T therapy	Chimera antigen receptor (CAR) T cell therapy changes the synthesis of platelet‐activating factors (PAFs)	Targeting PAF remodeling	^170^
Targeting immune checkpoint HLA‐E:CD94‐NKG2A	Immune checkpoint HLA‐E:CD94‐NKG2A on the tumor cells and T cell	Inhibiting the immune checkpoint	^69^
R848‐loaded nanoparticle	Coated with platelet membrane, which contained resiquimod (R848) and the drug	Agonist of toll‐like receptor	^192^
Nanoparticle with apatiniband and VDAs	Platelet membrane‐based nanoparticles loaded with apatinib, an antiangiogenic drug	Damage vessels and disrupt tumor angiogenesis	^193^
Glucose oxidase (GOx)‐based starvation therapy	Application of platelet membrane	Improve the efficacy of killing tumors	^194^
Mesoporous Fe single‐atom nanozyme (Fe‐SAzyme) coated with platelets	Platelet membrane used to camouflage single‐atom nanozyme	Destroy mitochondria	^196^
β‐Mangostin‐loaded nanoparticles	The hybrid membrane of both platelets and tumor cells used to coat the β‐mangostin‐loaded nanoparticles	Improved ability targeting glioma	^197^
Platelet exosomes	Produced by exosomes	FG@PEL to exert its cascade targeting effect against tumor	^198^
Platelet vesicles with RSL‐3	Platelet vesicles used to coat RSL‐3	Pass through the stroma and disrupt tumor angiogenesis	^199^
A nanoparticle coated with PSN peptide	Nanoparticles coated with a P‐selectin‐targeting peptide	Inhibiting platelet−tumor interaction and inflammation	^200^
Quercetin (QCT)‐loaded platelet	Drug to be loaded into the cytoplasm of platelets	Target the glioblastoma tumor	^201^
AuNR‐loaded platelets	Platelets loaded using AuNR	Target tumor cells precisely	^202^

Abbreviations: CART, chimera antigen receptor T cell therapy; FAK, focal adhesion kinase; Fe‐SAzyme, Fe single‐atom nanozyme; GOx, glucose oxidase; MMP‐2, matrix metalloprotein‐2; PDGFR, platelet‐derived growth factor; PTAFR, platelet‐activating factor receptor.

### 6.3 Current clinical trial progression

Nowadays, many current clinical trials about the function and regulation of platelets in cancer treatment have been in progression (Table 3). A research team values tumor‐educated platelets ITGA2B and selectin P (SELP) mRNA expression in pancreatic and biliary tract cancer. They take expression levels of ITGA2b and SELP on tumor‐educated platelets for diagnosis of pancreatic and biliary cancer as the standard to study the expression pattern of ITGA2b and SELP genes of tumor‐educated platelets in pancreatic and biliary tree cancer and its diagnostic value. Xing et al.^182^ have found that TEP ITGA2B is an important marker to improve the recognition of patients with stage I NSCLC and differentiate malignant from benign lung nodules. D'Ambrosi et al.^203^ indicate that further research on the RNA transfer mechanism between platelets and cancer cells can help us better understand and monitor the progression of cancer. What is more, the study showed the effects of inhibiting platelet function on circulating cancer cells for breast cancer patients. The purpose of this study is to determine the effects of Plavix and aspirin in women with metastatic breast cancer, and now the study is in phase 2. Platelet inhibition of CTCs is measured by the number of patients with detectable CTCs.^203^ Elevated PC as prognostic factor in colorectal cancer with synchronous liver metastases is also a new study. The aim of this study was to evaluate the role of preoperative PC in patients with synchronous colorectal liver metastases. Pedrazzani et al.^204^ research found that high PC values are significantly associated with overall survival and cancer‐related survival in TNM stage IV patients.

**TABLE 3:** Current status of academic research on the function and regulation of platelets in cancer therapy.

Interventions	Clinical registries	Status	Study results	Conditions	Study start	Study completion	Reference
Diagnostic test: mRNA expression	NCT05493878	Not yet recruiting	No results available	Pancreatic and biliary tract cancer	2023‐03 (estimated)	2024‐05 (estimated)	None
Diagnostic test: mRNA expression	GZR2017‐186	Completed	No results available	Non‐small‐cell lung cancer	Not reported	2017 (actual)	^182^
Biological: A single 2 mL heparinized peripheral blood	NCT02758678	Completed	No results available	Lung cancer	2015‐11 (actual)	2016‐10 (actual)	None
Biological: Blood samples containing K2‐EDTA	42763‐CRINF‐1034 CESC	Completed	No results available	Colorectal cancer		Not reported	^204^
Drug: Plavix Drug: Aspirin	NCT00263211	Terminated	Has results	Breast neoplasms	2006‐01 (actual)	2009‐09 (actual)	None
Diagnostic test: Platelet count	NCT02211677	Recruiting	No results available	Nasopharyngeal carcinoma	2014‐08 (actual)	2024‐12 (estimated)	None
Drug: Pembrolizumab Drug: Clopidogrel Drug: Acetylsalicylic acid	NCT03245489	Recruiting	No results available	Recurrent or metastatic squamous cell carcinoma of the head and neck	2017‐10‐20 (actual)	2023‐12‐31 (estimated)	None

Some researches evaluate the expression of P selectin on platelets of blood between serum samples from lung cancer and healthy individuals through serum separation and Raman spectroscopy analysis. Assessment of the concentration of P‐selectin in three groups in lung cancer patients and comparing the concentration of P‐selectin depends on tumor volume. Comparisons were made between the three groups and finally with the healthy population. Bendas and Borsig point out that integrins were shown to contribute to cancer progression.^205^

Best et al.^179^ characterize the platelet mRNA profiles of various crowds. They provide powerful evidence for the clinical relevance of blood platelets for liquid biopsy‐based molecular diagnostics in patients with different types of cancer.

## 7 CONCLUSION

**FIGURE 7:**
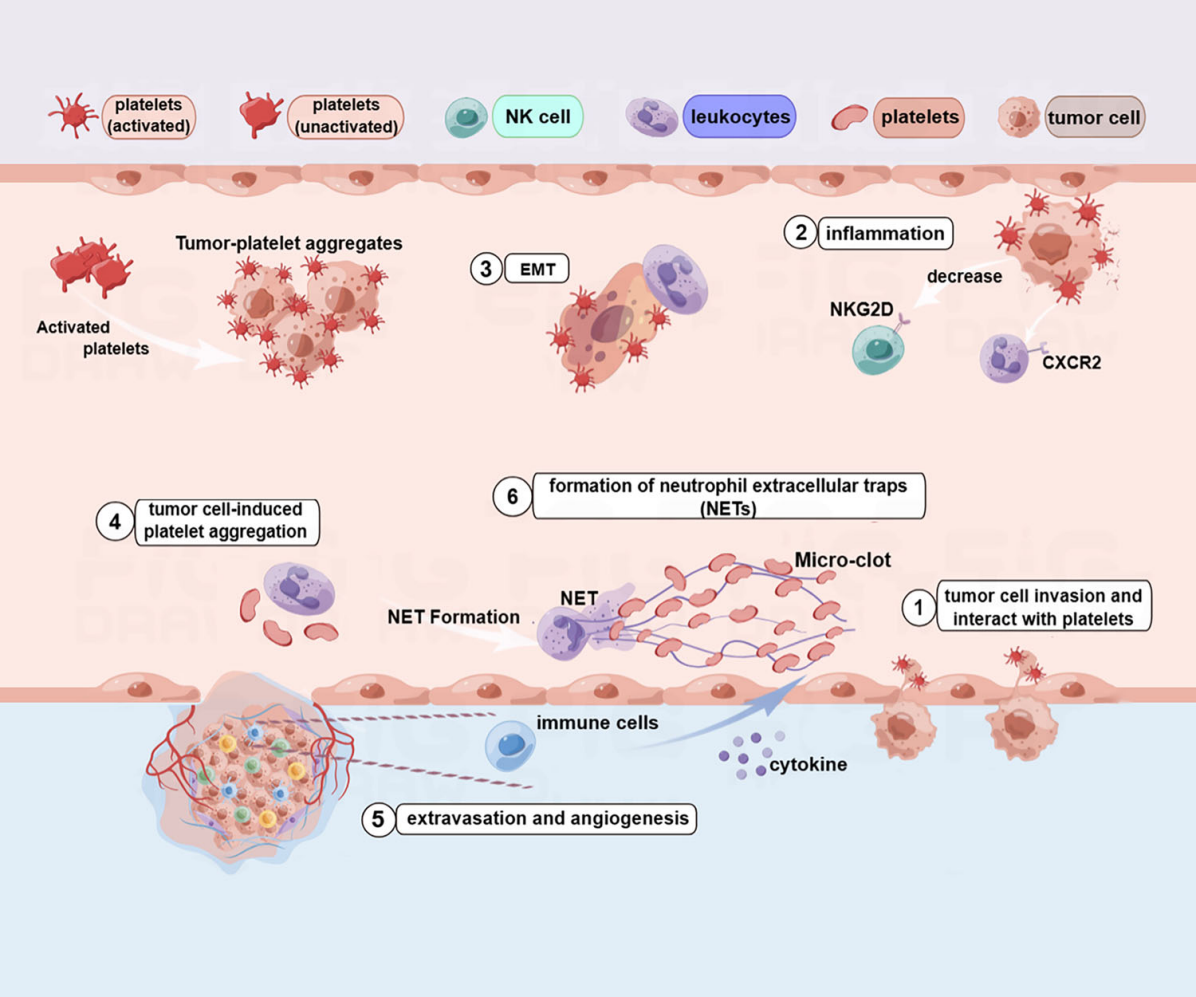
The summary of interactions between tumor cells and platelets. ① Platelets can facilitate the tumor cells invasion. ② The interaction can cause inflammation, causing the decrease of NKG2D on the surface of NK cells. ③ Leukocytes help the EMT. ④ In addition, tumor cells can cause platelet aggregation. ⑤ These can help the tumor cells extravasation and angiogenesis. ⑥ In the blood vessels, neutrophil extracellular traps (NETs) can stimulate the migration and invasion of cancer cells. Immune cells in the tumor microenvironment can release cytokines to promote the growth of cancer. The figure is made using Figdraw.

With the in‐depth study of platelet function, the hypothesis that platelets regulate the TME to promote tumor cell proliferation and metastasis through various ways has been gradually confirmed. This includes the formation of TCIPA to assist tumor cell immune escape and promotion of neovascularization leading to tumor cell extravasation (Figure 7). In addition, platelets are involved in tumor growth and metastasis through various mechanisms.For example, platelets releasing a large amount of active substances can interact with tumor cells leading to the invasion and metastasis of tumor cells. Compared with the large amount of evidence that platelets promote tumor metastasis, some experiments have found that platelets can inhibit tumor metastasis. This process is affected by many factors, providing new clues for the treatment of tumor metastasis. However, the specific mechanism is still unclear, and further studies about the relationship between platelets and tumors are worthwhile in the future, so as to prevent the tumor metastasis and progression.

Currently, there are clinical observations on the effects of antiplatelet drugs on recurrence and metastasis in tumor patients. Focusing on the platelets, the decrease of platelets can promote treatment. Antiplatelet drugs, such as aspirin (pro‐oxygenase inhibitors), can contribute to the prevention, occurrence, and recurrence of tumors, especially liver disease. It may be due to the fact that the liver is the main organ producing coagulation proteins. However, it remains questionable whether the antitumor effect of these drugs is the result of platelet inhibition, and drug resistance research is also an urgent question that needs to be addressed. Targeting the molecules or specific proteins, such as enzymes, provides effective ideas for cancer treatment. In addition, disturbing any process in the interaction between platelets and tumor cells is useful, such as angiogenesis, immune escape, and so on. More in‐depth and detailed studies are needed because platelets are involved in every process of tumor hematogenous metastasis, and factors, such as treatment time, dose, tumor site, and type, need to be considered. Nowadays, platelets assisting tumor cell invasion and metastasis are receiving increasing attention from researchers and can be a key direction and area for future treatment of oncological diseases.

This review summarized the interactions between platelets and tumors and furtherexplained the potential mechanisms of tumor cell homing and growth, tumor cell metastasis (including immune escape, angiogenesis, EMT), TME, and other processes regulating tumor development. What is more, the role and significance of platelets as cancer biomarkers is also pointed out. On this basis, we outlined the application of platelet‐based targeted therapeutic strategies in tumor treatment, and discussed the research field and corresponding development applications. In the future, more and more experiments will be required to validate the antitumor effects. According to the characteristics of platelets, it is possible to explore more drugs. In addition, platelets can combine with other antitumor therapies, which may be a direction in cancer therapies.

## AUTHOR CONTRIBUTIONS

Kaili Liao, Xue Zhang, and Wenyige Zhang wrote the main manuscript text and Qijun Yang, Feifei Teng, Yingcheng He, , Jie Liu, Daixin Guo, Gaoquan Cao, Yanmei Xu,Bo Huang and Yuxuan Xie searched the literature and prepared Figures 1−7. Jie Liu and Jinting Cheng participated in the writing and manuscript revision. Xiaozhong Wang reviewed and revised the manuscript and wrote guidance. All authors have read and approved the final manuscript.

## CONFLICT OF INTEREST STATEMENT

There are no conflict of interest in this study.

## ETHICAL APPROVAL AND CONSENT TO PARTICIPATE

This article does not contain any studies with patients or animals performed by any of the authors.

## Data Availability

All data are available. Please contact us to access if it is needed.
